# Advancements in Plasma-Enhanced Chemical Vapor Deposition for Producing Vertical Graphene Nanowalls

**DOI:** 10.3390/nano13182533

**Published:** 2023-09-11

**Authors:** Enric Bertran-Serra, Shahadev Rodriguez-Miguel, Zhuo Li, Yang Ma, Ghulam Farid, Stefanos Chaitoglou, Roger Amade, Rogelio Ospina, José-Luis Andújar

**Affiliations:** 1 ENPHOCAMAT (FEMAN) Group, Department of Applied Physics, Universitat de Barcelona, Martí i Franquès 1, E-08028 Barcelona, Spainstefanoschaitoglou@ub.edu (S.C.); r.amade@ub.edu (R.A.); rospinao@ub.edu (R.O.); jandujar@ub.edu (J.-L.A.); 2 Institute of Nanoscience and Nanotechnology (IN2UB), Universitat de Barcelona, E-08028 Barcelona, Spain; 3 Escuela de Física, Universidad Industrial de Santander, Carrera 27 Calle 9 Ciudad Universitaria, Bucaramanga 680002, Colombia

**Keywords:** graphene, carbon nanowalls (CNWs), vertical graphene nanowalls (VGNWs), plasma-enhanced chemical vapor deposition (PECVD), structural and morphological characteristics

## Abstract

In recent years, vertical graphene nanowalls (VGNWs) have gained significant attention due to their exceptional properties, including their high specific surface area, excellent electrical conductivity, scalability, and compatibility with transition metal compounds. These attributes position VGNWs as a compelling choice for various applications, such as energy storage, catalysis, and sensing, driving interest in their integration into next-generation commercial graphene-based devices. Among the diverse graphene synthesis methods, plasma-enhanced chemical vapor deposition (PECVD) stands out for its ability to create large-scale graphene films and VGNWs on diverse substrates. However, despite progress in optimizing the growth conditions to achieve micrometer-sized graphene nanowalls, a comprehensive understanding of the underlying physicochemical mechanisms that govern nanostructure formation remains elusive. Specifically, a deeper exploration of nanometric-level phenomena like nucleation, carbon precursor adsorption, and adatom surface diffusion is crucial for gaining precise control over the growth process. Hydrogen’s dual role as a co-catalyst and etchant in VGNW growth requires further investigation. This review aims to fill the knowledge gaps by investigating VGNW nucleation and growth using PECVD, with a focus on the impact of the temperature on the growth ratio and nucleation density across a broad temperature range. By providing insights into the PECVD process, this review aims to optimize the growth conditions for tailoring VGNW properties, facilitating applications in the fields of energy storage, catalysis, and sensing.

## 1. Introduction

Carbon nanowalls (CNWs) have attracted considerable interest in recent years, owing to their exceptional properties, including their high surface area and excellent conductivity, making them promising candidates for diverse applications. Significant progress in the field has been driven by numerous studies investigating different synthesis methods, growth conditions, and substrate materials to optimize CNWs’ properties. Among these, plasma-enhanced chemical vapor deposition (PECVD) has emerged as a prominent technique for producing large-scale graphene films and creating vertically oriented carbon nanowalls (VOCNWs) on various substrates. Overall, the methodologies employed for graphene growth and analysis have shown remarkable diversity. Different techniques with varying parameters have contributed to the understanding of graphene growth and the synthesis of unique carbon nanostructures like carbon nanowalls. This diversity has opened exciting opportunities for tailoring VGNWs’ properties for specific applications in energy storage, catalysis, and sensing.

As the field of PECVD for producing vertical graphene nanowalls (VGNWs) continues to evolve, a comprehensive review of the latest advancements is essential. By shedding light on the underlying physicochemical mechanisms that govern VGNWs’ formation and growth, this review aims to pave the way for further breakthroughs in this promising area of research and to accelerate the practical utilization of VGNWs in cutting-edge technologies. The insights gained from this review will serve as a valuable resource for researchers and engineers seeking to optimize the growth conditions and to harness the full potential of VGNWs for a wide range of applications.

Earlier reviews by Vesel et al. [1] and Hiramatsu and Hori [2] provided comprehensive overviews of the achievements in CNWs deposition, establishing a foundation for subsequent research endeavors. Wu et al. [3] presented the first report on CNWs synthesis using a methane and hydrogen gas mixture, highlighting the role of the substrate temperature and catalyst in the growth process. Since then, various studies have explored alternative methods, such as capacitive coupled plasma chemical vapor deposition (CCP-PECVD) and microwave plasma-enhanced chemical vapor deposition (MWCVD), resulting in high growth rates and morphological variations. Crucial investigations have emphasized the significance of hydrogen in gas mixtures, the competition between growth and etching, and the role of Ar in CNWs growth.

The field of carbon nanomaterials has witnessed remarkable advancements, exemplified by the pioneering work of Zhang et al. [4], showcasing accelerated growth rates and novel morphological structures. These advancements have unlocked new opportunities for the applications of carbon nanowalls (CNWs) across various domains. However, to fully harness the potential of CNWs, a comprehensive understanding of their underlying growth mechanisms is imperative. This review is motivated by the necessity to delve deeper into these growth mechanisms and to explore the advancements in the synthesis of vertical graphene nanowalls (VGNWs) using plasma-enhanced chemical vapor deposition (PECVD). The primary objectives of this review encompass investigating the influence of the temperature on the number of atomic layers in VGNWs and gaining insights into critical parameters, such as the plasma conditions, gas flow, and pressure, that dictate the growth process. By unraveling the intricate physicochemical mechanisms governing the formation of VGNWs, this review seeks to facilitate the establishment of optimized growth conditions tailored to specific applications in energy storage, catalysis, and sensing.

The landscape of graphene research has been significantly enriched by a diverse range of methodologies employed for growth and analysis, leading to an enhanced comprehension of CNWs’ synthesis and unique carbon nanostructures. Various techniques—including chemical vapor deposition (CVD) growth, hot filament CVD (HFCVD) [5], direct current (DC) arc discharge evaporation [6], PECVD, and other techniques evolved from it such as inductively-coupled PECVD (ICP-CVD) [7,8], remote PECVD (RPECVD) [7], plasma-enhanced atomic layer deposition (PEALD) [9], or other more specific like high-voltage nanosecond pulses for radical injection (RI)-PECVD [10]—have been effectively utilized with varying parameters, yielding distinct morphologies and properties. Crucially, characterization techniques such as scanning electron microscopy (SEM), transmission electron microscopy (TEM), Raman spectroscopy, and X-ray photoelectron spectroscopy (XPS) have played pivotal roles in offering essential insights into the structural and property characteristics of various carbon nanostructures.

Given the substantial progress in the field and the specific promise demonstrated by VGNWs in diverse applications, a comprehensive and systematic review of the advancements in PECVD-based synthesis and growth mechanisms is of paramount importance. By bridging the gaps in the current knowledge and providing an in-depth understanding of the growth process, this review aims to accelerate the development and practical implementation of VGNWs in cutting-edge technologies.

The evolution of graphene growth experiments has engendered the development of a varied spectrum of experimental setups and procedures. Notably, Chaitoglou et al. [11] explored graphene nucleation through CVD growth at varying temperatures, estimating an activation energy of 3.01 eV. While prior studies have predominantly focused on multi-layered graphene, the present study directs its focus towards investigating the impact of the growth temperature on the number of atomic layers in VGNWs. Specifically, the utilization of inductively coupled plasma-enhanced chemical vapor deposition (ICP-CVD) with methane as the carbon precursor is examined, elucidating the essential parameters that steer the growth process, including the plasma conditions, gas flow, and pressure. The intrinsic merits of ICP-CVD, such as the elimination of catalysts and the heightened electron density of the plasma, are emphasized.

Additional methodologies have also significantly contributed to the body of knowledge in the realm of CNWs. Ando et al. [6] employed the DC arc-discharge evaporation of graphite in a hydrogen-filled vacuum chamber, yielding interlaced petal-like sheets and nanometric petal-like structures. Exploiting the manipulation of graphene’s electronic structure through surface defect sites and functional-group modifications, researchers have successfully enhanced its electrocatalytic activity. Notably, Wang et al. [12] reported the development of three-dimensional graphene networks with abundant sharp edge sites through the deposition of vertical graphene sheets on SiOx nanowire networks, showcasing their excellent electrocatalytic activity for hydrogen evolution reaction (HER). Furthermore, PECVD-synthesized carbon nanowalls have been thoroughly examined for their electrochemical properties by Lehmann et al. [13], revealing insights into their morphology, nanostructure, defects in the graphitic lattice, and the electrochemically active surface area.

A notable innovation lies in the synthesis of 3D graphene networks, exemplified by the work of He et al. [14]. Their fabrication involves pressing commercially available Ni foam, subsequent cleaning, and graphene coating using chemical vapor deposition, resulting in a flexible, mechanically robust, and highly electrically conductive 3D graphene network. The electrodeposition of MnO_2_ onto these networks yields a graphene/MnO_2_ composite with desirable capacitance and electrochemical performance, rendering it suitable for flexible electrode applications.

The appeal of vertical graphene as a nanocarbon thin-film material lies in its unique 3D hierarchical structures, as highlighted by Hang et al. [15] and Zheng et al. [16]. In contrast to planar graphene growth, vertical graphene presents abundant out-exposing edges, high porosity, nano passages, and a substantial surface area, making it an ideal candidate for applications in electrochemistry, bioelectronics, and flexible electronics. However, the exploration of vertical graphene comes with its share of challenges, including nomenclature standardization, complex structure characterization, growth mechanism understanding, and integrating small-sample processing into mass production. Despite these challenges, the potential of vertical graphene extends to diverse applications such as flexible electronics, microenergy conversion, electrocatalysis, biosensing, and wearable devices, surpassing conventional graphene and carbon nanotube materials. Notably, the water splitting process through electrocatalysis offers a promising avenue for producing clean and renewable molecular hydrogen (H_2_) fuel.

Recent progress has seen the successful growth of VGNWs on flexible stainless-steel substrates, rendering them suitable for integration into electrochemical systems like batteries, supercapacitors, and catalysts [7]. While the growth of graphene nanowalls has historically focused on multi-layered graphene structures, this study delves into the effect of the temperature on the atomic layer count within VGNWs. The growth of high-quality bilayer VGNWs around 700 °C is presented, accompanied by comprehensive details of the growth conditions and analytical methodologies employed to scrutinize the structural and morphological features of these VGNWs. The use of ICP-CVD with methane as the carbon precursor is discussed, shedding light on the critical parameters for growth modulation, such as the plasma conditions, gas flow dynamics, and pressure. The advantageous attributes of ICP-CVD, including the absence of catalysts and enhanced electron density of the plasma, are highlighted. The study rigorously explores the impact of the growth temperature through Raman spectroscopy and electron microscopy, revealing that carbon nanostructures’ growth on SS310 substrates is profoundly influenced by the temperature. Capacitively-coupled plasma (CCP) is found to favor carbon nanotube (CNT) growth, while inductively coupled plasma (ICP) promotes the growth of VGNWs. The obtained insights provide a detailed understanding of the growth mechanisms of carbon nanostructures. The temperature range of 675 °C to 775 °C is identified as conducive for the growth of graphene nanowalls through ICP-CVD on SS310 substrates from pure methane without a catalyst, with the thickness of the graphene nanowalls being at least two monatomic layers. Notably, defects mainly reside at the nanowalls’ edges rather than their faces, enhancing their electric transport characteristics and facilitating the growth of metallic oxide and carbide nanoparticles for energy storage and catalyst applications.

In regard to VGNWS applications, we have selected the most recent advances in the most interesting areas today, such as, photocatalysis [17,18], electrocatalyst for highly efficient hydrogen evolution reaction (HER) [19,20,21], rechargeable battery technology [22,23,24], supercapacitor technology [7,25,26,27,28,29,30], hydrovoltaic power generation [31], solar energy conversion [32,33,34,35], efficient thermal interfaces [36,37,38,39], field emission [40], IR detectors [41], and gas sensing [5,42,43].

In summary, this review aims to consolidate the wealth of knowledge concerning the VGNW growth mechanisms and applications, fostering a comprehensive understanding that will guide future research efforts and expedite the real-world implementation of VGNWs across a wide range of domains.

## 2. Methods and Discussion

Recent advancements, exemplified by Zhang et al.’s work [4], have demonstrated faster growth rates and novel morphological structures, providing new opportunities for CNWs’ applications. However, to fully leverage their potential, a deeper understanding of the underlying growth mechanisms is imperative. In this context, the present review delves into the advancements in PECVD for producing vertical graphene nanowalls (VGNWs). The primary objectives are to explore the impact of temperature on the number of atomic layers in VGNWs and to gain insights into critical factors, like the plasma conditions, gas flow, and pressure, that are crucial for modulating the growth process. By understanding the physicochemical mechanisms steering VGNWs formation, this review seeks to facilitate optimized growth conditions tailored for specific applications in energy storage, catalysis, and sensing.

The diversity of the methodologies used for graphene growth and analysis has significantly contributed to the understanding of CNWs’ synthesis and unique carbon nanostructures (Table 1). Various techniques, including CVD growth, DC arc discharge evaporation, and PECVD, have been utilized with varying parameters, yielding distinct morphologies and properties. Crucial insights into the structures and properties of various carbon nanostructures have been unveiled through characterization techniques like scanning electron microscopy (SEM), transmission electron microscopy (TEM), Raman spectroscopy, and X-ray photoelectron spectroscopy (XPS).

Given the significant progress in the field, particularly the potential of VGNWs for diverse applications, a comprehensive review of the advancements in PECVD-based synthesis and growth mechanisms becomes indispensable. Such a review bridges the knowledge gaps, presents a holistic understanding of the growth process, and consequently accelerates the integration of VGNWs into cutting-edge technologies. Furthermore, the evolution of graphene growth experiments has led to the development of a diverse array of setups and procedures. Bertran and Chaitoglou [7,44] investigated graphene nucleation through CVD growth at varying temperatures, estimating an activation energy of 3.01 eV. The synthesis of carbon nanowalls has usually focused on multi-layered graphene; however, these studies aim to investigate the effect of the temperature on the number of atomic layers in VGNWs. The authors discuss the use of ICP-CVD with methane as the carbon precursor, providing insights into the crucial parameters for modulating the growth process, such as the plasma conditions, gas flow, and pressure. The advantages of ICP-CVD, such as the absence of catalysts for VGNWs nucleation, low ion energy, and higher electron density of the plasma, are highlighted.

Ando et al. [6] employed the DC arc-discharge evaporation of graphite in a hydrogen-filled vacuum chamber, resulting in interlaced petal-like sheets and smaller nanometric petal-like structures. More recent research has demonstrated the enhancement of graphene’s electrocatalytic activity via modifications to its electronic structure of graphene through surface defect sites and functional-group modifications. For instance, Wang et al. [12] developed three-dimensional graphene networks with abundant sharp edge sites by depositing vertical graphene sheets on SiO_x_ nanowire networks. The resulting 3D graphene networks exhibited excellent electrocatalytic activity for hydrogen evolution reaction (HER), making them highly active and stable metal-free and dopant-free HER electrocatalysts. Also, the study of the electrochemical activity of carbon nanowalls (CNWs) was examined through the morphology and nanostructure of CNWs synthesized through PECVD [13]. The height of the CNWs increased with the deposition time, forming interconnected vertically aligned carbon sheets. Characterization techniques, such as Raman spectroscopy, revealed defects in the graphitic lattice, and X-ray photoelectron spectroscopy (XPS) confirmed the presence of sp2 hybridized carbon and the absence of heteroatom doping. The electrochemically active surface area of the CNWs increased with the film thickness and deposition time. However, the peak potential difference in the cyclic voltammetry measurements was larger than expected, possibly due to surface contamination. These findings provide invaluable insights into the morphology and electrochemical properties of hierarchical carbon nanowalls (hCNWs) synthesized through PECVD. Lehmann et al. synthesized hCNWs using radio frequency PECVD with p-xylene as the carbon precursor [13]. Microwave reactors (MW) operating in the TE mode, like the one utilized by Bo et al. [45], have played a pivotal role in vertical graphene (VG) synthesis. Bo et al. utilized a 2.45 GHz MW source coupled to a cylindrical quartz tube to create a standing wave with the strongest electric field in the growth region. To overcome the limitations of transverse electric microwave (TE-MW) reactors, the transverse magnetic microwave (TM-MW) reactor was introduced, allowing better control of the substrate temperature, and enabling higher operating power and pressure. Notably, Hiramatsu et al. [8] grew carbon nanowalls (CNWs) via ICP-CVD using a CH_4_/Ar mixture, exploring the roles of various substrates (n-type Si(100) and SiO_2_-coated Si(100) substrates with a 50 nm oxide layer, along with Ti-catalyzed SiO_2_-coated Si substrates) in nucleation enhancement. Yu et al. [46] employed a plasma reactor operating at atmospheric pressure, utilizing argon as the plasma gas and diverse substrates like silicon wafers and stainless steel plates.

The journey of innovation also led to the creation of 3D graphene networks, where pressing commercially available Ni foam, cleaning it, and coating it with graphene via chemical vapor deposition yielded flexible, mechanically strong, and highly electrically conductive 3D graphene networks [14]. The pressed Ni foam prevents the network from collapsing and cracking during bending. These graphene networks, further modified through the electrodeposition of MnO_2_, result in a uniform coating over the entire surface. The networks of the graphene/MnO_2_ composite demonstrated a high capacitance and electrochemical performance, holding promise for flexible electrode applications.

In the pursuit of understanding vertical graphene, Hang et al. [15] unveiled its unique 3D hierarchical structures, offering potential applications in electrochemistry, bioelectronics, flexible electronics, and more, such as those reported in the recent review by Zheng et al. [16]. Unlike planar growth graphene, vertical graphene has abundant out-exposing edges, high porosity, nano-passages, and a large surface area, making it suitable for applications in electrochemistry, bioelectronics, and flexible electronics. However, the challenges of standardizing nomenclature, characterizing complex structures, understanding the growth mechanisms, and transitioning small-sample processing to mass production need to be addressed. The potential of vertical graphene spans areas like flexible electronics, microenergy conversion, electrocatalysis, biosensing, and wearable devices, superseding conventional graphene and carbon nanotube materials. Electrochemical water splitting for producing molecular hydrogen (H_2_) emerges as a promising avenue.

Recent strides have seen the growth of VGNWs on flexible stainless steel substrates suitable for electrochemical systems such as batteries, supercapacitors, and catalysts [7]. This study hones in on the effect of the temperature on the number of atomic layers in VGNWs. Notably, high quality bilayer vertical graphene nanowalls were grown at temperatures of around 700 °C. The experiment’s details encompass the growth conditions, methodology for structural and morphological analysis, and insights into the crucial parameters for modulating the growth process. The supremacy of ICP-CVD, marked by its catalyst-free nature and higher plasma electron density, is highlighted.

In this fundamental study, the impact of the growth temperature on the structure and morphology of carbon nanostructures, examined through Raman spectroscopy and electron microscopy, was investigated. Carbon nanostructures were grown on SS310 substrates at different temperatures using a remote plasma process.

It is important to underscore that within the context of ICP-CVD, a notable preference is observed for fostering the growth of VGNWs in contrast to carbon nanotubes (CNTs). Notably, the growth of VGNWs through ICP-CVD obviates the necessity for substrate activation through catalyst nanoparticles, such as Fe. This distinction is especially pertinent as the typical growth trajectory of carbon nanotubes invariably demands the presence of catalyst nanoparticles for the initiation and sustained progression of their growth process.

A significant observation reported in this study [7] is that, in the specific case of employing stainless steel substrates, the surface’s inherent iron content facilitates the coexistence of CNTs alongside VGNWs during the growth process. It is noteworthy, however, that unlike the growth processes realized through capacitively coupled plasma chemical vapor deposition (CCP-CVD), ICP-CVD processes exhibit a distinct behavior. Specifically, within ICP-CVD processes, the growth kinetics of VGNW structures demonstrate a superior growth rate in comparison to that of carbon nanotubes (CNTs).

This observation underscores the intrinsic disparities in the growth dynamics of carbon nanostructures achieved through distinct plasma-enhanced chemical vapor deposition methods. Such nuances further underscore the pivotal role that the plasma conditions, catalyst presence, and underlying substrate characteristics play in steering the growth trajectory of these nanostructures. The identification of the pivotal role of ICP-CVD in favoring VGNW growth over CNTs, while minimizing the reliance on catalyst nanoparticles, augments our understanding of the tailored synthesis strategies for diverse carbon nanostructures, thereby advancing our capacity to design and engineer materials for targeted applications.

**Figure 1:**
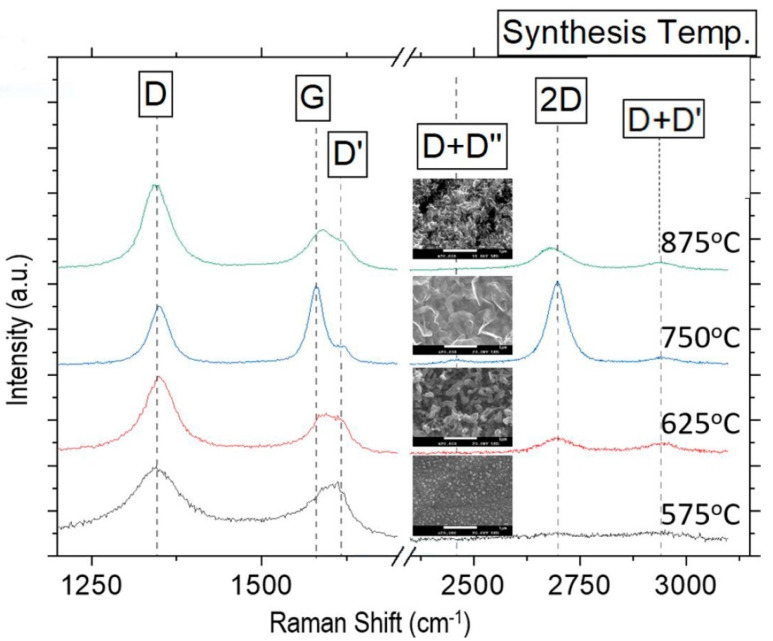
Normalized Raman spectra of VGNWs and carbon nanotubes (CNTs) grown on stainless-steel at different temperature. At temperatures around 750 °C, graphene nanowalls of sizes around 1 μm present the characteristic 2D peak of graphene and an *I_D_*/*I_G_* ratio lower than 1 indicating a lower number of defects [7]. (Reproduced with permission).

**Figure 2:**
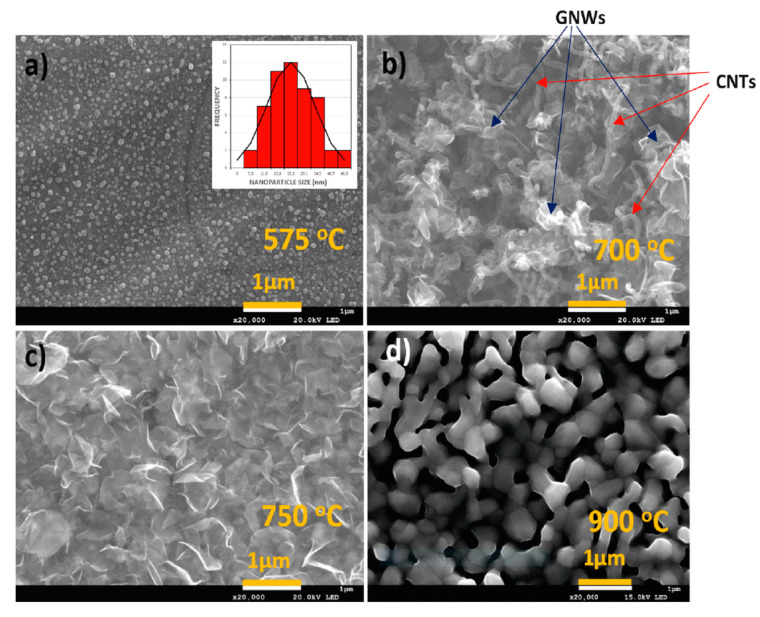
FE-SEM images of carbon nanostructures grown on stainless-steel (SS310) substrate by ICP-CVD from CH_4_, corresponding to different processing temperatures: (**a**) Nucleation of carbon nanostructures on iron domains from the substrate at 575 °C. (**b**) Growth of a mixture of carbon nanotubes (CNTs) and graphene nanowalls (GNWs) at 700 °C. (**c**) Growth of GNWs of dimensions around 1 µm at 750 °C. (**d**) Formation of Cr particles segregated from the stainless-steel substrate (SS310) and their coalescence. Carbon nanostructures are not formed at 900 °C [7] (Reproduced with permission).

**Figure 3:**
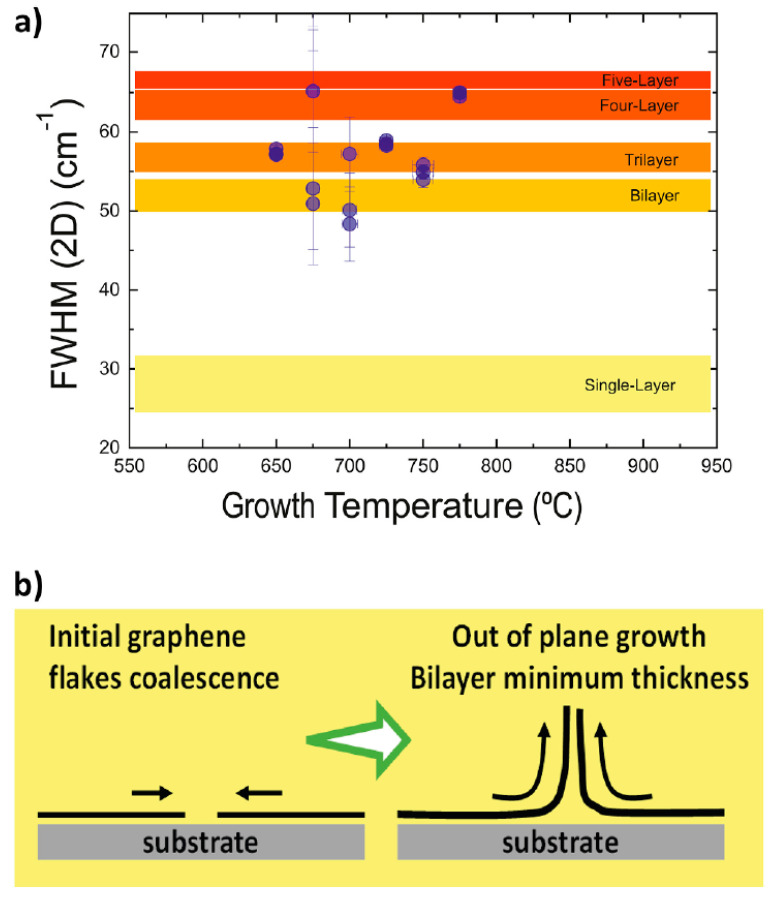
Plot of FWHM of 2D Raman peak versus growth temperature of VGNW’s prepared by ICP-CVD. (**a**) In colored bands there is a correspondence between FWHM (2D) and the number of monoatomic graphene layers. (**b**) Schematic representation of the process of formation of VGNWs growing from the edges. This process gives rise to the formation of graphene nanowalls that protrude from the surface of the substrate or by nucleation from CNTs, in seemingly random directions around the normal one to the substrate [7] (Reproduced with permission).

Raman spectroscopy was used to analyze the carbon nanostructures, confirming the presence of graphene nanowalls and carbon nanotubes at different growth temperatures (Figure 1). The study provided valuable insights into the growth mechanisms of carbon nanostructures (Figure 2). This study concludes that the temperature range for the ICP-CVD growth of graphene nanowalls on SS310 substrates using pure methane and without a catalyst spans 675 °C to 775 °C. The characteristics resulting from the analysis using Raman spectroscopy of the graphene nanowalls show that their thickness is at least two monoatomic layers as a result of the growth mechanism of the graphene nanowalls in 3D formations (Figure 3). Also, the defects in the graphene nanowalls in the above-mentioned temperature range reside mainly on the nanowalls’ edges—and not on their faces—which upgrades their electric transport characteristics and affects and facilitates the growth in a prominent place of nanoparticles of metallic oxides and carbides (MeO_x_ and MeC_y_) for energy storage and catalysts applications.

Another very interesting paper on graphene nanowalls formation is the work of Baranov et al. [47], which details the formation mechanism of carbon nanostructures using a multi-scale, multi-factor model validated using experimental data. Kondo et al. [48] investigated the initial growth process of CNWs on Si substrates via radical injection PECVD with O_2_ gas added to the C_2_F_6_/H_2_ source gas, effectively controlling CNW nucleation. Giese et al. [49] studied the synthesis and characterization of CNWs on diverse substrates, identifying four distinct morphologies of CNWs and proposing a growth mechanism based on surface diffusion and particle energies.

Further advances come from Khan et al. [50], who leveraged conventional high-temperature direct thermal CVD and the plasma-enhanced CVD (PECVD) of graphene on cubic-silicon carbide (3C-SiC) surfaces to fabricate high-quality hetero-junctions. Notably, they successfully grew controlled and self-limiting layers of 3C-SiC on Si(100) substrates via thermal CVD to obtain virtual 3C-SiC substrates. However, due to the negligible carbon atom diffusion over 3C-SiC, graphene growth did not occur. Nonetheless, they successfully grew graphene nanowalls (GNWs) on both Si and 3C-SiC/Si surfaces at 700 °C through PECVD, as the growth mechanism of GNWs is independent of the substrate type. The performance of GNW/SiC/Si heterostructures in terms of current conduction significantly outperformed SiC/Si heterostructures, showcasing the potential of GNWs in enhancing device performance.

Notably, Cong et al. [9] demonstrated the growth of large-area graphene nanowalls (GNWs) on Si substrates via PEALD, paving the way for high-performance GNWs-Si heterostructure infrared photodetectors (PDs). The process utilized benzene as the carbon source and formic acid for the GNW growth. Sun et al. [51] explored vertically oriented graphene (VG) growth on GaN nanowire tips, discovering intriguing phenomena, where VG grows along the field direction, perpendicular to the particles’ local surfaces. These insights open doors for further vertical two-dimensional material growth mechanism studies.

Recently, Chaitoglou et al. [19] demonstrated that GNWs are the vertical derivatives of 2-dimensional graphene, growing them on various metallic and non-metallic substrates, providing valuable insights into their morphology and crystalline quality. The inherent electrocatalytic properties of GNWs towards HER in an acidic medium were evaluated for all substrates, showcasing the potential of GNWs for metal-free electro-catalytic electrodes, replacing rare or noble metals in diverse practical applications.

The importance of understanding these underlying mechanisms cannot be overstated, as it paves the way for precise control over the growth process, leading to tailored properties of VGNWs for specific applications. By elucidating the role of hydrogen in graphene growth and investigating the dual functionalities of hydrogen as a co-catalyst and etchant for nanowalls formation, this review contributes to a deeper comprehension of the intricate growth process of VGNWs.

Moreover, the exploration of various growth methodologies, such as CVD, DC arc discharge evaporation, microwave reactors, plasma-enhanced chemical vapor deposition and its variations (RPECVD, ICPCVD), and PEALD, has led to a diverse toolkit for producing carbon nanowalls. This diversity opens exciting possibilities for customizing VGNWs to suit different application requirements.

The potential of VGNWs as a versatile material for energy storage, catalysis, and sensing applications is tremendous. The advancements in growth techniques, morphological variations, and the understanding of the nucleation processes have propelled this field forward. However, the journey does not end here. Further research is needed to unravel the full potential of VGNWs and to optimize their properties for practical applications.

**Table 1:** Methods and Conditions of VGNWs Growth.

Ref./Year	Gas	Temperature (°C)	Pressure	Technology Frequency	Power (W)	Coating/Substrate	Main Conclusions
[23]/2019	CH_4_/H_2_	625	400 mTorr	RF-PECVD (13.56 MHz)	20–80	VGNWs/Ge<111>	VAGNAs can be used as an efficient SERS substrate
[7]/2023	CH_4_	575 to 900	400 mTorr	ICP-CVD (13.56 MHz)	400	VGNWs/Stainless-steel SS310	Impact of temperature on morphology and structure of VGNWs
[52]/2020	Ar/H_2_/C_2_H_2_	700	10–150 Pa	PECVD	300	VGNWs/SiO_2_, SiO_2_/Ti, SiO_2_/Ti/Pt	cross-section micrograph about 18 μm, width of edges less than 10 nm.
[19]/2022	CH_4_	750	400 mTorr	ICP-CVD (13.56 MHz)	440	VGNWs/Stainless-steel SS310, Polycrystalline-Cu,Papiex©	growth of VGNWs in a variety of metallic and non-metallic substrates insights on morphology and crystalline quality
[21]/2023	CH_4_	750	400 mTorr	ICP-CVD (13.56 MHz)	400	Mo_2_C/Papiex©	VGNWs as template with abundant defects favoring bonding of ns-Mo2C
[24]/2020	CTAB/deionized water	200 (24 h)	-	hydrothermal process	-	MoS_2_@rGO	Fabrication of MoS2@rGO nanowall structure
[25]/2016	CH_4_/N_2_ + CH_4_	-	20 mTorr to 760 Torr	MW plasma torch (MPT) (2.45 GHz)/PECVD	500–1500	GNW/Ti NGNW/Ti	GNW/Ti and NGNW/Ti electrodes extend upper potential limit of a positive electrode of EDLCs from 0.1 V to 1.3–1.5 V
[9]/2021	-	-	-	PEALD	-	GNWs/Si	GNW-Si Schottky junction-based selfpowered IR PD with high responsivity
[53]/2015	C_2_H_2_/Ar/H_2_	550–750	200–400 Pa	PECVD	150	CNWs/SiC	field emission properties of the CNWs
[34]/2023	-	-	-	PECVD	-	VGNWs/textured c-Si	PEDOT doped textured VGNWs/Si Schottky junction
[49]/2018	Al acetylacetonate	350–425–500	8 Pa	ICP-PECVD	500	CNWs	CNWs morphologies depending on process
[40]/2019	Glucose/ureaAr	850	70 kPa	Spin-coating/CVD	-	N:VGNs/304SS	growing intrinsic and nitrogen-doped VGNs on stainless steel
[15]/2019	C precursor	-	-	MW-PECVD/ALD	-	VGNWs/ZnO nanorods	Hierarchical Graphene/Nanorods-Based H_2_O_2_ Electrochemical Sensor
[28]/2020	H_2_/C_2_H_4_	450–620	29 Pa	CC-PECVD 13.56 MHz	-	VGNWs	Growth VGNWs by CC-PECVD at low temperature (450 °C), using Ni catalyst
[10]/2020	CH_4_/H_2_	650	-	(ns)-RI-PECVD	400	CNWs	isolated carbon nanowalls via high-voltage ns pulses (ns)-RI-PECVD
[54]/2013	CH_4_/H_2_	-	-	ICPCVD	-	VGNWs	Synthesis of VGNWs for field emitters
[50]/2022	C precursor	700	-	PECVD	-	VGNWs/c-Si VGNWs/3C-SiC	VGNWs/SiC interfacial layers for heterojunction devices
[48]/2009	C_2_H_6_/H_2_	930	160 Pa	RI-PECVD 2.45 GHz	250–270	CNWs/Si,SiO_2_,Al_2_O_3_,Ni	CNWs growth by RI-PECVD
[55]/2022	C precursor	450	-	PECVD	-	VGNWs	VGNWs growth at low temperature plasma
[43]/2021	C precursor	600	500 mTorr	MW-PECVD	1300	CNWs/SiO_2_/p-Si	CNWs/SiO_2_/Si gas sensor
[13]/2018	p-xylene	415	4.7 Pa	ICPCVD	150	Hierarchical CNW	hCNW synthesized by a PECVD
[17]/2020	-	-	-	-	-	(Li_3_O)_n_,(Na_3_O)_n_,(K_3_O)_n_ @GDY	Design of Graphdiyne-based materials for optoelectronic applications
[35]/2023	C precursor + Nafion	-	-	-	-	VGNWs/Si	VGNWs/Si Schottky junction solar cells with Nafion doping
[41]/2020	Ar/H_2_/CH_4_	800	7 Pa	PECVD	200	VGNWs/VO_2_(B)	VGNWs/VO_2_(B phase) for IR detector
[5]/2023	PDMS	400	-	HF-CVD	-	VGNWs	VGNWs for flexible pressure sensor
[56]/2020	CH_4_	750	50 to 60 Pa	ICP-CVD	440	CNSs/SS304	Photoluminescence from CNSs
[27]/2019	PAN+DMF CH_4_/H_2_	600	600 Pa	Electrospinning MW-PECVD	350	G-CNFs	G-CNFs-MnO_2_ electrodes for supercapacitors
[33]/2020	CH_4_/H_2_	750	-	PECVD	50	VGNH/Si	VGNHs/c-Si Shottky junction solar cells
[22]/2018	Ar/H_2_/CH_4_	1050	800 Pa	Mesoplasma, MPCVD	10 kW	VGN/Ni@Li foam	VGN/Ni@Li foam for pseudocapacitance induced fast Li+ ion transfer
[26]/2017	Ar/CH_4_	800	-	(ECR)-PECVD	375	VGNWs/Ni	VGNWs/Ni for supercapacitor application
[51]/2022	C_2_H_2_	-	-	PECVD	-	VGNWs/GaN-NWVGNWs/np-SiO_2_	Growth of VGNWs/GaN-NW and VGNWs/np-SiO_2_ by PECVD
[57]/2020	Ar	350	14.5 Pa	PECVD 13.56 MHz	500	np-Pt/CNWs	synthesis of Pt/CNW sheet electrocatalysts
[58]/2017	Ar/H_2_/C_2_H_2_	700	10 to 150 Pa	Ar plasma jet	-	CNWs	wettability of plasma deposited CNWs
[37]/2022	C_2_H_2_	150	-	HF-CVD	-	VGNWs	Synthesis of VGNWs on dielectrics
[42]/2019	gaseous camphor	600	30 Pa	CVD	-	Graphene/ZnO/Graphene	Graphene/ZnO-NWs/Graphene Heterojunction for NO_2_ Gas Sensor
[38]/2022	ChloroformC precursor	650	-	Electric field assisted PECVD	250	VG arrays	Rapid growth of VG arrays for TIM
[36]/2020	methane, ethanol, methanol	650	-	AEF-PECVD	250	VG arrays/Cu, glass, c-Si	Vertical Graphene Arrays for TIM
[39]/2023	C precursor	-	-	PECVD	-	VGNs/CF/ss	VGNWs/C fibers for TIM
[59]/2019	Ar/H_2_/CH_4_	750–900	-	CC-PECVD	550–770	VGNWs	VGNWs for Li-ion batteries
[60]/2023	C precursor	-	-	RF and RI-PECVD	-	CNWs/Al_2_O_3_ nanopores	Creation of CNWs/Al_2_O_3_ nanopores
[61]/2021	Ar/CH_4_	800	-	ICP-PECVD	140	CNWs	Properties of CNWs
[31]/2022	C precursor	-	500 Pa	HF-CVD	-	VGNWs/substrate	VGNWs for hydrovoltaic power generation
[62]/2023	C precursor	-	-	CVD	-	ns-G/W/dielectric	Multimode THz absorber based on ns-G
[63]/2023	C precursor	-	-	CVD on Cu catalyst	-	SLG/SiO_2_/Au	SLG/SiO_2_/Au for absorber on SPR
[64]/2022	-	-	-	-	-	PIT/ns-G/dielectric subst.	Theoretical study of PIT/ns-G/substrate
[65]/2023	C precursor	-	-	CVD on Cu catalyst	-	SLG/SiO_2_/Au	SLG/SiO_2_/Au for THz absorber on SPR

## 3. Growth Mechanism of VGNWs

Significant contributions have been made to the understanding of graphene nanowall formation, including the comprehensive multi-factor model by Baranov et al. [47], which was validated through experimental data. In this study, they concluded that the ion bombardment is the reason for the VGNWs’ growth in the mechanism when the building blocks are supplied through the surface diffusion, but not directly from the gas phase. The assumption of the surface diffusion of hydrocarbon radicals as a dominant mechanism of VGNWs growth is consistent with the existing experimental data [7,19]. The main findings of the study by Baranov et al. are: Ion bombardment energy is sufficient to activate the surface by generating defects and exciting the adsorbed species, thus ensuring the conditions for fast nanoflake growth. Nanowalls do not grow through gas phase deposition, but mainly from the substrate surface under conditions of ion bombardment without any catalyst on the surface. Kondo et al. [48] investigated the initial growth process of carbon nanowalls on Si substrates using radical injection PECVD with added O_2_ gas, effectively controlling the nucleation. Giese et al. [49] identified distinct CNW morphologies on various substrates and proposed a growth mechanism based on surface diffusion and particle energies.

Moreover, Cong et al. [9] introduced a novel approach to growing large-area graphene nanowalls (GNWs) on Si substrates using plasma-enhanced atomic layer deposition (PEALD), yielding high-performance GNWs-Si heterostructure infrared photodetectors. Sun et al. [51] explored the growth of vertically oriented graphene (VG) on GaN nanowire tips, demonstrating unique growth phenomenology on round-shaped nanoparticles. Khan et al. [50] explored graphene growth on cubic silicon carbide (3C-SiC) surfaces using thermal and plasma-enhanced CVD, yielding insights into hetero-junctions and enhancing the current conduction.

Further contributions from Chaitoglou et al. [19] revealed GNWs as vertical derivatives of 2D graphene and highlighted their inherent electrocatalytic properties toward hydrogen evolution reaction (HER) in an acidic medium.

**Figure 4:**
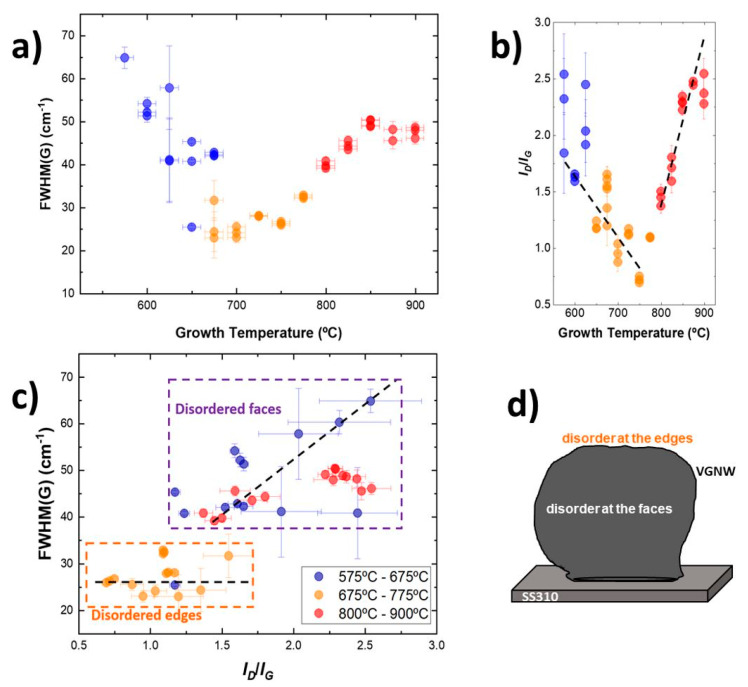
Quality of the graphene nanowalls as a function of the growth temperature on SS310 stainless steel [7]. (**a**) Evolution of the FWHM of the G peak with growth temperature, with a minimum in the temperature range of 650 °C to 750 °C. (**b**) Plot of *I_D_*/*I_G_* versus the growth temperature pointing to an opposite behavior of samples grown at lower temperature (blue and amber points) and the ones grown at high temperatures (red points). (**c**) Plot representing the FWHM(G) versus the intensity ratio *I_D_*/*I_G_*. In the case of disorder located at the edges, FWHM(G) is not correlated with *I_D_*/*I_G_* (flat dashed line) for points inside the bottom-left rectangle, which correspond to the graphene nanowalls having between 2 and 4 atomic layers. (**d**) Schematic representation of a scale of VGNW grown on SS310 substrate (Reproduced with permission).

As an example of the morphology of VGNWs from the growing process [7], in Figure 4, we have illustrated the consequences of the Evolution of the FWHM of the G peak with the growth temperature—with a minimum in the temperature range of 650 °C to 750 °C—in the Plot of *I_D_*/*I_G_* versus the growth temperature, pointing to oppositional behavior of the samples grown at lower temperatures and the ones grown at high temperatures. In the case of disorder located at the edges, *FWHM*(*I_G_*) is not correlated with *I_D_*/*I_G_*, which correspond to the graphene nanowalls with between two and four atomic layers. A schematic representation of a scale of VGNW grown on SS310 substrate is illustrated in Figure 4d.

The importance of comprehending these intricate mechanisms cannot be overstated as it enables precise control over the growth process and the tailoring of the VGNW properties for specific applications. Investigating the role of hydrogen in graphene growth and understanding its dual functions as a co-catalyst and etchant in nanowall formation deepens our grasp of the VGNW growth process.

The diversity of the growth methodologies—mainly including CVD, DC arc discharge evaporation, microwave reactors, PEALD, PECVD, and other techniques like ICP-CVD, and RPECVD—underscores the dynamic nature of CNW, and specifically VGNWs synthesis. The potential of VGNWs as versatile materials for energy storage, catalysis, and sensing applications is both substantial and promising. The amalgamation of growth technique advancements, morphological variations, and nucleation insights propels the field forward. However, this journey is far from complete, as ongoing research endeavors aim to uncover the complete spectrum of VGNWs’ potential and optimize their properties for diverse practical applications.

## 4. Applications of VGNWs

### 4.1. Advancements in Photocatalysis Using Graphdiyne

Graphdiyne (GDY), a distinctive two-dimensional carbon allotrope, has garnered significant attention for its exceptional properties and potential applications. Inexpensive and possessing a high performance, GDY exhibits a combination of sp and sp2 hybridized carbon atoms, forming a planar structure with versatile properties. Synthesized by Li and co-workers in 2010 [17], GDY boasts diacetylene linkages, a conjugated system, wide surface spacing, porosity distribution, tunable electronic properties, chemical stability, and semiconductor characteristics. Tigges et al. [57] present a comprehensive review examining GDY’s attributes, synthesis techniques, bandgap tunability, and recent advances in photocatalytic applications. Various structural morphologies, such as nanotubes, nanowires, nanosheets, nanowalls, and 3D frameworks, are discussed, along with the theoretical analyses that initially characterized GDY. Importantly, experimental validations have been reported, bolstering the feasibility of GDY-based photocatalytic applications.

The review underscores the extensive exploration of GDY-based nanocomposites for photodegradation, the photoreduction of CO_2_, and photocatalytic hydrogen production, positioning GDY as a key player in the next generation of carbon-derived photocatalytic systems.

Shandilya et al. [18] offer a similar perspective, emphasizing GDY’s potential as a versatile and high-performance nanomaterial. The synthesis, properties, and tunable bandgap of GDY are explored alongside recent advancements in its application within photocatalysis. The authors discuss the exploration of various structural morphologies, from nanotubes to 3D frameworks. The experimental validation of GDY’s theoretical potential in photocatalytic contexts is highlighted, with an extensive investigation into photodegradation, the photoreduction of CO_2_, and photocatalytic hydrogen production.

### 4.2. Electrocatalyst for Highly Efficient Hydrogen Evolution Reaction

Efficient and sustainable hydrogen production is a critical pursuit, driving the exploration of novel electrocatalysts that are both highly efficient and cost-effective. Notably, Chaitoglou and colleagues [19,20,21] have made significant strides in this field, ushering in a new era of electrocatalysis for the hydrogen evolution reaction (HER). In their study [20], the team presents a groundbreaking approach involving a nanostructured Mo carbide film synthesized via chemical vapor deposition. This innovative catalyst exhibits remarkable electrocatalytic activity for HER in acidic media. The interconnection between the carbide film and the underlying Mo foil results in exceptional performance metrics, including a Tafel slope of −65 mV/dec and an overpotential of 330 mV at 10 mA/cm^2^. Notably, the catalyst maintains its excellent durability even after 1000 cycles. An even more remarkable advancement is showcased when a graphene/Mo_2_C heterostructure is simultaneously grown, forming a vertical stack. This heterostructure displays Tafel slopes and overpotentials akin to commercially available Pt catalysts, a pivotal step towards precious metal-free catalysis.

In a subsequent work [19], the inherent properties of vertical graphene flakes, also known as graphene nanowalls (GNWs), are probed for their electrocatalytic activity towards HER. The team demonstrates that when GNWs are strategically deposited, they either enhance or exhibit inherent catalytic activity depending on the electrode’s intrinsic behavior. This versatility is further validated through successful deposition on flexible electrodes, with the GNWs showing impressive stability, even after manual bending. This study pioneers the insight into the inherent electrocatalytic activity of GNWs, advocating their potential for novel catalyst development. Further amplifying their contributions, Chaitoglou et al. [21] present a hybrid nanostructured approach, combining Mo_2_C with vertical graphene nanowalls. This ingenious synergy is achieved through chemical vapor deposition, magnetron sputtering, and thermal annealing. The resultant compounds display remarkable activities for HER in acidic media, marked by impressively low overpotentials and Tafel slopes, highlighting the potential of such hybrid catalysts for sustainable hydrogen production.

### 4.3. Rechargeable Battery Technology

The pursuit of high-performance rechargeable batteries has led to groundbreaking research in the field, showcasing significant advancements in design and materials. Ren et al. [22] address the challenge of uneven dendrite growth in lithium (Li) metal anodes, a key obstacle in high-capacity applications. Their novel approach involves a 3D vertical graphene nanowalls on nickel foam (VGN/Ni) structure, which exhibits pseudo-capacitive interfacial features that enhance the Li+ ion transfer kinetics. This innovation enables uniform Li plating and stripping, leading to stable Li metal cycling, even at high depths of discharge. The resulting symmetrical cells, with VGN/Ni@Li composite anodes, showcase remarkable Coulombic efficiencies and stability over extended cycling, setting the stage for high-energy-density batteries. In a related work, Yang et al. [59] explore VGNWs as anode materials in Li-ion batteries. They demonstrate the tailored growth of VGNWs through varying experimental conditions, revealing the growth mechanism of plasma-based synthesis. These VGNWs exhibit exceptional cycle efficiency and specific capacity, presenting a promising avenue for carbon-based nanomaterial applications and a potential advancement in battery performance.

Al-Hagri et al. [23] introduced vertically aligned graphene nanosheet arrays (VAGNAs) grown on germanium using PECVD without a catalyst. These VAGNAs, terminated with a high-quality single-layer graphene sheet, hold immense potential in various applications due to their large surface area, electron transport properties, and electrochemical activity. Their remarkable properties pave the way for applications in batteries, supercapacitors, and beyond, offering enhanced sensitivity for surface-enhanced Raman spectroscopy.

Chen et al. [24] tackle the challenges of MoS_2_ in sodium-ion batteries (SIBs), namely their low rate capability and poor cycle stability. They developed a unique composite by growing vertically-oriented MoS_2_ on reduced graphene oxide (rGO) nanowalls using electrostatic attraction. This design not only enhances the specific surface area and active site exposure, but also improves the electronic conductivity and structural stability. The resulting MoS_2_@rGO composite displays an impressive electrochemical performance, maintaining capacity over numerous cycles and even at ultra-high current densities. This innovation holds promise for large-scale SIB applications.

Collectively, these studies denote significant advancements in rechargeable battery technology, addressing the critical challenges and demonstrating the potential for more efficient, stable, and high-performance energy storage solutions.

### 4.4. Innovations in Supercapacitor Technology

The field of supercapacitors is experiencing remarkable advancements in design and material strategies, fueling their potential to revolutionize energy storage applications. Chi et al. [25] highlight the integration of electrical double-layer capacitors (EDLCs) as a means to enhancing energy storage systems. They propose the concept of “choosing a matching pair of electrode materials and electrolytes” to extend the cell voltage of EDLCs. This strategy involves using inert-surface materials, like vertically grown graphene nanowalls (GNWs), to expand the upper and lower potential limits of electrode materials, resulting in an asymmetric EDLC with a cell voltage of 4 V, impressive energy density, and outstanding stability. An additional innovation was proposed by Sahoo et al. [26], addressing the synthesis of vertical graphene nanosheets (VGNs) for flexible devices. They introduced a polymer-free transfer technique for VGNs onto arbitrary substrates, preserving their morphology, structure, and properties. The transferred VGNs enable the creation of binder-free and current collector-free flexible symmetric supercapacitors with excellent capacitance and cycle retention, emphasizing the potential of VGNs in flexible nanoelectronic devices. Later, Qi et al. [27] explored the combination of electric double-layer capacitance and pseudo-capacitance to enhance the supercapacitor performance. They designed hierarchical microstructured electrodes by integrating MnO_2_ nanoparticles on plasma-grown vertically oriented graphenes (VGs). This unique electrode structure exhibits high capacitance and stability, suggesting new ways to improve the electrochemical capacitor performance. Also, Hussain et al. [28] tackled the challenge of the low-temperature synthesis of vertical graphene nanosheets (VGNs) for energy applications. They developed a controlled synthesis method using capacitively coupled plasma at low temperatures and power, facilitated by nickel (Ni) catalysts. This approach enhances the growth rate, density, and quality of VGNs, expanding the potential for practical VGN applications.

Zhang et al. [29] emphasized the significance of the electrode structure in supercapacitors. They highlight the advantages of three-dimensional vertically aligned graphene (3DVAG) electrodes, which offer excellent reaction kinetics, mass transfer capability, and energy storage performance.

The review published by Sahoo et al. [30] discusses the preparation methods, application areas, and challenges in utilizing 3DVAG materials, providing insights into their potential for high-energy-density electrode materials.

Bertran-Serra et al. [7] focused on the growth of vertical graphene nanowalls (VGNWs) on flexible substrates for electrochemical applications. They highlighted the use of 3D carbon nanostructures as growth templates for electrochemically active materials, enabling large specific surface areas. Their study investigates the effect of the growth temperature on the morphological and structural characteristics of VGNWs, showcasing the potential of VGNWs for high-performance supercapacitors.

These studies underline the rapid progress and potential of supercapacitors, offering innovative strategies to enhance the energy storage performance through advanced electrode materials, tailored synthesis methods, and optimized electrode structures.

### 4.5. Unlocking Hydrovoltaic Power Generation with VGNWs

Hydrovoltaic power generation—harnessing the energy of water droplets—has gained significant attention due to its potential for sustainable energy harvesting. Zhu et al. [31] present a breakthrough in this field by successfully preparing high-quality vertical graphene nanowalls (VGN) through the hot-wire chemical vapor deposition (HWCVD) method, eliminating the need for catalysts. The researchers achieved a defect-free VGN with few layers and submicron domain sizes by finely tuning the growth conditions. Optimal VGN quality was confirmed through Raman spectroscopy, showcasing an I-D/I-G value of less than 1 and an I-2D/I-G exceeding 2.8. The deposition pressure was identified as a critical parameter influencing the crystallization quality, with 500 Pa pressure facilitating the growth of high-quality VGN. Remarkably, the VGN exhibited excellent electrical properties and was incorporated into hydrovoltaic power generation devices. The VGN’s enhanced crystal domain area and reduced contact angle contributed to its outstanding performance in energy harvesting. At a deposition pressure of 100 Pa, the VGN-based hydrovoltaic device achieved a remarkable maximum output power of 15.7 µW, surpassing the previous reported values in this field. This study establishes VGN as a promising candidate for nanoscale energy harvesting applications, emphasizing its potential to drive innovation in hydrovoltaic power generators.

### 4.6. Advancements in Solar Energy Conversion Using VGNWs

Efficient solar energy conversion is a pivotal pursuit in the realm of renewable energy. Graphene—with its exceptional photothermal properties and broadband absorption capabilities—and VGNW/n-Si heterojunctions for photovoltaic conversion have emerged as promising candidates for solar–thermal energy conversion. Recent research has showcased innovative strategies utilizing vertical graphene nanowalls (VGNWs) to enhance solar energy conversion efficiency, paving the way for practical applications.

Ren et al. [32] introduced a groundbreaking approach by developing hierarchical graphene foam (h-G foam) through plasma-enhanced chemical vapor deposition. This unique structure demonstrated remarkable omnidirectional and broadband sunlight absorption, enabling a substantial elevation of the temperature. Utilizing h-G foam as a heating material, an impressive external solar–thermal energy conversion efficiency of approximately 93.4% was achieved. Notably, the solar–vapor conversion efficiency exceeded 90% for applications like seawater desalination, highlighting the potential of graphene-based materials for addressing pressing global challenges.

In another endeavor, Rehman et al. [33] demonstrated the viability of VGNWs for solar cell applications. They grew VGNWs directly on an interfacial layer of Al_2_O_3_, minimizing surface recombination and enhancing the built-in potential. Through careful optimization of the VGNW thickness and silicon surface texturing, they overcame the challenges related to high silicon reflectivity. By co-doping with PEDOT: PSS and inorganic acid HNO_3_, they achieved a high conversion efficiency of 10.97%, along with a noteworthy photo-responsivity under deep ultraviolet light. This research signifies the potential of VGNWs as a platform for efficient solar cell technology.

Cui et al. [34] addressed the industrialization of graphene/silicon solar cells by preparing large-area VGNWs on textured silicon substrates. They overcame the challenges associated with insulation films by introducing conductive-passivating PEDOT:Nafion composite thin films. Additionally, the incorporation of an Al2O3 interfacial layer led to an impressive 11.75% efficiency in VGNWs/Si solar cells, with enhanced light management and reduced reflectivity. This work contributes to the advancement of VGNWs for practical solar cell applications.

Liu et al. [35] extended the utility of VGNWs in vertical graphene nanowall/silicon Schottky junction solar cells by doping them with polymeric acid (Nafion) and employing plasma etching. The innovative combination of Nafion doping and plasma etching significantly improved the carrier transport and enhanced the VGNW/n-Si heterojunction’s properties. This approach resulted in a notable power conversion efficiency of 9.2% for VGNW/n-Si solar cells. The device architecture and optimization scheme proposed in this research offer valuable insights for enhancing the performance of other 3D materials with similar structures.

### 4.7. Efficient Thermal Interfaces for Enhanced Electronic Device Performance

As electronic devices push the boundaries of power density, miniaturization, and multifunctionality, the significance of thermal interface materials (TIMs) becomes paramount. The quest for effective thermal management has led to breakthroughs in the field of thermal interfaces, catalyzing advancements in electronic device performance. Xu et al. [36] addressed the challenge of high-quality vertical graphene (VG) array fabrication by harnessing an alcohol-based electric-field-assisted PECVD method. This innovative approach leverages alcohol-based carbon sources to augment the growth rates and mitigate defects. The alignment of graphene sheets via a vertical electric field results in high-quality, vertically aligned graphene, reaching a height of 18.7 μm. The TIMs constructed using VG arrays display remarkable characteristics: a vertical thermal conductivity of 53.5 W m^−1^·K^−1^ and a low contact thermal resistance of 11.8 K·mm^2^·W^−1^. This demonstrates the potential of VG arrays in heat dissipation technologies. Subsequently, Wang et al. [37] revolutionized VG growth by realizing near room-temperature VGN growth using catalytic Ta filaments through HF-CVD. The ability to grow VGNs directly on substrates below 150 °C offers exceptional interfacial contact, reducing the electronic chip temperatures by 6.7 °C compared to conventional thermal conductive tape.

Xu et al. [38] introduced a chloroform-assisted method for the rapid growth of VG arrays, yielding heights of up to 100 μm. These VG arrays, when integrated into a composite TIM, exhibit a remarkable vertical thermal conductivity of 34.2 W·m⁻^1^·K⁻^1^, significantly enhancing thermal dissipation in light-emitting diodes. Recently, Yan et al. [39] presented an innovative approach to heat dissipation with a novel vertical graphene nanosheets/carbon fibers (VGNs/CF) composite film. By combining carbon fibers and VGNs, they created a continuous thermally conductive network, resulting in a through-plane thermal conductivity of 17.7 W/(m·K), surpassing the performance of pure VGN film.

These pioneering studies collectively underscore the transformative potential of thermal interfaces in enhancing the performance and reliability of electronic devices. The adoption of innovative materials and fabrication techniques promises a future of efficient and effective thermal management in the electronics industry.

### 4.8. Advancing Field Emission Technology through Vertical Graphene Nanosheets

VGNs have emerged as a versatile material with immense potential in various applications, including field electron emitters. In their pursuit of enhancing field emission technology, Guo et al. [40] presented a groundbreaking approach that not only simplifies the growth of VGNs, but also optimizes their performance. In this study, the researchers introduce an innovative and eco-friendly method for growing both intrinsic and nitrogen-doped VGNs on stainless steel (SS) substrates. The process involves heating thin layers of glucose and/or urea in a resistance-heating furnace. Notably, the growth of VGNs predominantly occurs on roughened regions of the SS substrate, attributed to the higher concentration of nucleation and catalyzing sites in these areas compared to smoother regions. Significant advantages of this technique are its scalability and cost-effectiveness, offering promising avenues for practical applications. By adjusting the addition of urea, the nitrogen doping concentration within the VGNs can be finely tuned. Field emission measurements revealed the exceptional capabilities of the resulting nitrogen-doped VGNs. These findings underscore the transformative potential of nitrogen-doped VGNs as efficient and reliable field electron emitters. Their research not only simplifies the growth process of VGNs, but also demonstrates the viability of these materials in real-world applications, thus contributing to the advancement of field emission technology.

### 4.9. IR Detectors with VGNWs/VO_2_ Composite Films

Infrared detectors play a crucial role in various applications, and their performance largely depends on the materials used. Vanadium dioxide (VO_2_) stands out as a superior choice due to its exceptional thermal sensitivity, especially in its B phase. However, conventional VO_2_ (B) films face challenges like a low temperature-coefficient of resistance (TCR) values and high resistances. In their groundbreaking study, Lu et al. [41] presented a remarkable solution by introducing composite films of VGNWs/VO_2_ (B). Leveraging a low-pressure chemical vapor deposition process, they successfully fabricated these composite films with a unique blend of properties. The resulting films exhibited a carefully controlled square resistance of 12.98 kΩ and an improved temperature-coefficient of resistance (TCR) of −3.2% per degree Kelvin. The secret to the enhanced performance lies in the unique advantages of vertical graphene nanowalls. These nanowalls provide an efficient channel for electron transport, significantly enhancing the conductivity of VO_2_ (B) films. This breakthrough holds promise for the design and creation of high-performance VO_2_ (B) thin films, revolutionizing the field of uncooled infrared detectors. This study not only addresses the limitations of traditional VO_2_ (B) films, but also demonstrates the potential of composite materials to drive advancements in infrared detection technology. The carefully engineered VGNWs/VO_2_ (B) composite films pave the way for more efficient and effective infrared detectors, opening doors to a wide range of applications.

### 4.10. Photonic Devices Based on Graphene Nanostructures

Among the photonic devices, terahertz (THz) absorbers have attracted significant attention due to their broad range of applications in fields such as photodetectors, optoelectronic devices, chemical sensors, and electromagnetic shielding. Graphene nanostructures have emerged as promising candidates for achieving efficient THz absorption due to their unique electronic and optical properties. Recent developments in the utilization of graphene-based nanostructures for THz absorbers have been reported by Chen et al. [65], Lai et al. [63], Tang et al. [64], and Ye et al. [62].

Graphene—a single layer of carbon atoms arranged in a honeycomb lattice—possesses exceptional electrical conductivity, mechanical strength, and optical properties. These characteristics make it an ideal candidate for various applications, including THz absorbers. In recent years, researchers have explored novel designs and structures to enhance the THz absorption efficiency using graphene-based materials. Chen et al. [65] introduced a triple-band metamaterial absorber based on surface plasmon resonance (SPR). This absorber offers remarkable features, including perfect three-mode absorption, polarization independence, incident angle insensitivity, tunability, and a high figure of merit (FOM). It comprises a sandwiched stack with a top layer of single-layer graphene featuring an open-ended prohibited sign type (OPST) pattern, a middle layer of SiO_2_, and a bottom layer of gold metal mirror (Au). The absorber demonstrated perfect absorption at three distinct frequencies (4.04 THz, 6.76 THz, and 9.40 THz) and exhibited high sensitivity and tunability. This design holds promise for applications in photodetectors, active optoelectronic devices, and chemical sensors. Lai et al. [63] proposed a multi-frequency broadband absorber that relies on graphene SPR. By utilizing patterned graphene surface plasmon resonance, they achieved broad-band absorption bands ranging between 4.14 THz and 8.66 THz. Through the superposition of absorbing units responding to different frequency bands, they achieved perfect ultra-wideband absorption. This design showcases the potential applications in terahertz photoelectric detection, filtering, and electromagnetic shielding. Tang et al. [64] explored polarization-controlled and symmetry-dependent multiple plasmon-induced transparency (PIT) in a graphene-based metasurface. Their unit cell design consisted of reversely placed U-shaped graphene nanostructures and a rectangular graphene ring on a dielectric substrate. By adjusting the incident light’s polarization and nanostructure symmetry, they actively modulated the number of transparency windows, offering potential applications in mid-infrared optoelectronic devices. Ye et al. [62] introduced a tunable absorber based on graphene with a tunable Fermi level. The proposed absorber achieved two perfect absorption peaks in its working band (90–155 μm) with high efficiency. The researchers demonstrated the ability to adjust the absorption frequency by controlling the relaxation time and Fermi level of the graphene, as well as by changing the refractive index of the medium. This absorber’s flexibility in adapting to different electromagnetic environments offers possibilities for various practical applications.

### 4.11. Gas Sensing with Innovative Heterojunctions and Carbon Nanowalls

Gas sensors play a pivotal role in environmental monitoring and safety, and recent advancements have brought about exciting breakthroughs in this field. Xiong et al. [42] presented a pioneering approach by fabricating an upstanding graphene/ZnO/graphene sandwich heterojunction for room temperature NO_2_ detection. This novel structure combines the strengths of few-layer graphene’s electrical conductivity with ZnO nanowalls’ high sensitivity and selectivity towards NO_2_. The resulting sensor not only offers a rapid response recovery time, but also exhibits excellent selectivity against various other gases, indicating the potential for further enhancements through the integration of additional 2D materials. Shifting the focus to carbon-based gas sensors, Kwon et al. [43] explored the remarkable potential of carbon nanowalls (CNWs) synthesized via plasma-enhanced chemical vapor deposition. These CNWs exhibited a porous nanostructure with unique surface defects. The team demonstrated the distinct sensitivity of CNWs-based gas sensors to ammonia (NH_3_) and nitrogen dioxide (NO_2_), which are both known environmental pollutants. The sensor’s impressive performance in detecting nitrogen dioxide arises from the charge transfer differences and varying electron affinities, offering new avenues for gas sensor applications involving CNWs. In another exciting development, Mao et al. [5] harnessed the power of vertical graphene nanowalls (VGNs) to create flexible pressure sensors. VGNs, prepared using HF-CVD, possess outstanding electrical conductivity and specific surface area. These properties are harnessed in a flexible sensor, integrated with a polydimethylsiloxane (PDMS) elastomer. The result is a sensor with high sensitivity, swift response and recovery times, and the unique capability to adjust the sensitivity by controlling the VGN thickness. This innovation holds tremendous promise in human motion detection and pressure measurement applications.

## 5. Future Challenges

Despite the remarkable progress in the field of PECVD for producing CNWs, there are still several challenges and areas of improvement to address.

(a) Catalyst-Free Growth: Tanaka et al. [66] reported the catalyst-free growth of CNWs, which simplifies the fabrication process. However, further investigations are required to optimize this approach and to understand the factors that influence catalyst-free growth. Eliminating the need for catalysts can reduce costs and simplify the overall production process.
(b) Control of Morphology: While recent studies have explored different morphological forms of CNWs, achieving precise control over their structure remains a challenge. The understanding of how the process parameters influence the growth and morphology of CNWs is essential for tailoring their properties for specific applications.
(c) Uniformity and Scalability: The uniformity of CNWs over large areas is crucial for their practical applications. As the demand for CNWs in industrial and commercial settings increases, scalability becomes a vital consideration. Developing techniques that can ensure uniform and large-scale CNWs production is necessary for their widespread implementation.
(d) Characterization and Standardization: As the field advances, it is essential to establish standardized characterization techniques to accurately evaluate the quality, structure, and properties of CNWs. Standardization will facilitate comparison between studies and accelerate the progress in this area.
(e) Surface and Interface Engineering: CNWs’ surface and interface engineering is crucial for tailoring their properties for specific applications. By functionalizing or doping CNWs, their electrical, mechanical, and chemical characteristics can be tuned to meet the requirements of various devices and technologies.
(f) Integration with Devices: For practical applications, CNWs need to be seamlessly integrated with various electronic and optoelectronic devices. Research on the compatibility and effective integration of CNWs into existing device architectures is essential for realizing their potential in real-world applications.
(g) Cost-Effectiveness: As with any new technology, cost-effectiveness plays a crucial role in determining its commercial viability. Finding more cost-efficient synthesis methods, optimizing precursor gases, and improving the deposition rates will be key factors in making CNWs commercially competitive.

## 6. Conclusions

In the field of PECVD for generating VGNWs, significant strides have been achieved since its inception in 2002. Researchers have diligently explored an array of growth techniques, gas mixtures, conditions, and substrates, all with the aim of optimizing carbon nanowall synthesis. This multifaceted research landscape has given rise to novel carbon nanostructures with great potential in energy storage, catalysis, and sensing applications. This review paper focuses on the substantial strides made in PECVD for VGNW synthesis. It delves into the pivotal nucleation and coalescence stages, elucidating hydrogen’s critical role and the impact of the temperature on the graphene growth ratio and nucleation density. By offering a comprehensive overview of the recent developments in VGNWs—particularly their nucleation and growth through PECVD—this review encompasses various substrates, growth techniques, and characterization methods. This sheds light on the VGNW growth mechanisms and properties, enhancing our grasp of the PECVD process. This, in turn, empowers researchers to optimize the growth conditions for tailoring the VGNW properties for energy storage, catalysis, and sensing applications.

An especially noteworthy finding revolves around the effect of the temperature on the graphene growth ratio and nucleation density, observed across a wide temperature range. This insight not only enriches our understanding of the growth process, but also unveils the physicochemical mechanisms underlying VGNW formation. In regard to its applications, the exceptional potential of graphene-derived materials (GDY) in photocatalysis stands out. With a tunable bandgap, unique properties, and evolving validation, GDY hold promise in addressing environmental and energy challenges. Notably, advancements in electrocatalysis for hydrogen evolution reaction (HER) applications, such as nanostructured Mo carbide films and graphene-based hybrid catalysts, offer a sustainable path for hydrogen production and energy conversion technologies. The integration of VGNWs into solar energy conversion applications showcases impressive strides, promising high-efficiency solar energy conversion and propelling renewable energy technologies. The pioneering thermal interface materials (TIMs) studies highlight their transformative potential in enhancing electronic device performance and reliability, paving the way for efficient thermal management. Within the field of gas and pressure sensing, the reviewed studies underscore the transformative potential of novel materials and designs. From graphene-based heterojunctions to carbon nanowalls and flexible pressure sensors, these breakthroughs promise safer and more efficient gas sensing technologies.

Graphene nanostructures have emerged as versatile platforms for designing efficient THz absorbers with a wide range of applications. The studies discussed herein highlight the potential of graphene-based absorbers in achieving multi-band absorption, tunability, and sensitivity, making them promising candidates for various fields, including photonics, optoelectronics, and sensing technologies.

This review aimed to inspire the pursuit of VGNWs’ myriad possibilities, fostering transformative impacts in scientific and industrial applications.

## Data Availability

Not applicable.
